# Imaging-detected bone stress injuries at the Tokyo 2020 summer Olympics: epidemiology, injury onset, and competition withdrawal rate

**DOI:** 10.1186/s12891-022-05725-8

**Published:** 2022-08-10

**Authors:** Takuya Adachi, Hiroki Katagiri, Jae-Sung An, Lars Engebretsen, Ukihide Tateishi, Yukihisa Saida, Hideyuki Koga, Kazuyoshi Yagishita, Kentaro Onishi, Bruce B. Forster

**Affiliations:** 1 Department of Diagnostic Radiology, Tokyo Medical and Dental University, 1-5-45Bunkyo-Ku, YushimaTokyo, 113-8510 Japan; 2 Department of Joint Surgery and Sports Medicine, Tokyo Medical and Dental University, Tokyo, Japan; 3 Orthopedic Clinic, Oslo University Hospital and Oslo Sports Trauma Research Center, Norwegian School of Sport Sciences, Oslo, Norway; 4 Clinical Center for Sports Medicine and Sports Dentistry, Tokyo Medical and Dental University, Tokyo, Japan; 5 Department of Physical Medicine and Rehabilitation, University of Pittsburgh, Pittsburgh, PA USA; 6 Department of Radiology, Faculty of Medicine, University of British Columbia, Vancouver, Canada

**Keywords:** Fractures, Stress, Olympics, Magnetic resonance imaging, Radiology, Athletes

## Abstract

**Background:**

Prevention and early detection of injuries are essential in optimising sport participation and performance. The aim of this study is to investigate the epidemiology, athlete injury history, and competition withdrawal rate of imaging-detected bone stress injuries during the Tokyo 2020 Olympic Games.

**Methods:**

We collected and analysed imaging and clinical information in athletes with bone stress injuries diagnosed in the Olympic Village polyclinic during the Games. Two physicians independently and retrospectively reviewed all imaging examinations of bone stress injuries.

**Results:**

A total of 11,315 individual athletes from 206 National Olympic Committees competed at the Games, during which 567 MRIs and 352 X-rays were performed at the Olympic Village polyclinic. Radiology examinations revealed four stress fractures and 38 stress reactions in 29 athletes (median age 24 years, range 18–35 years). Of these, 72% of athletes (*n* = 21) had symptoms before entering the Olympic Village. Bone stress injuries were most common in women (55%), the lower extremities (66%), and track and field athletes (45%). Six athletes (21%) did not start or did not finish their competitions.

**Conclusions:**

This study revealed 42 imaging-detected bone stress injuries in the polyclinic of the Tokyo 2020 Olympic Village. The high proportion of athletes with symptoms before entering the village and the high proportion of competition withdrawals suggests the usefulness of an early MRI examination.

## Background

Bone stress injury, a common injury in athletes of all ages and skill levels, is an overuse injury associated with repetitive and intense loading of the bone due to increased volume or intensity of training workload. It does not accompany any specific episode of trauma and is therefore distinguished from traumatic fractures. Bone stress injury is generally classified into two stages: stress reaction and stress fracture. Stress reaction shows periostitis or bone marrow oedema on Magnetic Resonance Imaging (MRI) or nuclear medicine bone scan. Although stress reaction does not yet have cortical disruption or abnormal findings on X-ray, it can develop into a stress fracture if untreated. Stress fracture is an advanced stage of bone stress injury and can demonstrate periosteal reaction and ultimately a cortical disruption on X-ray, CT, and MRI. Prevention and early detection of bone stress injuries are essential in optimising sport participation and performance. Under some circumstances, stress fractures may even require a more extended treatment than typical traumatic fractures [1]. It has also been reported that a higher magnetic resonance imaging (MRI) grade of the stress fracture can delay return to competition [2, 3], and that 21% of stress fractures are recurrent, with 20% resulting in season-ending injuries [4]. In addition, known risk factors of bone stress injuries increase the risk for osteoporosis, a significant long-term health concern [5].

Previous studies of bone stress injury at the Olympic Games were conducted at Rio de Janeiro (2016) and London (2012) [6–12], where they accounted for approximately 2–3% of all injuries [13]. In these summer Olympics, bone stress injuries were most common in female athletes, track and field athletes, and the lower extremities [13, 14]. Except for these two Olympic Games, most epidemiological reports of injuries in past Olympics or competitions with similarly elite athletes have uniformly reported traumatic and stress fractures without distinction [5–9], and therefore a detailed epidemiological analysis focused on bone stress injuries in international competitions is lacking.

Furthermore, previous reports detailing bone stress injuries in the Olympic Games have not correlated imaging findings with medical history because of the lack of connectivity between Picture Archiving and Communication System (PACS) and Electronic Medical Records (EMR). Such connectivity was established for Tokyo 2020, allowing this correlation and aiding in understanding the clinical impact of these injuries. Therefore, the present study aims to investigate the epidemiology, athlete injury history, and clinical impact, including competition withdrawal rate, of imaging-detected bone stress injuries during the Tokyo 2020 Olympic Games.

## Methods

This retrospective study was approved by the ethics committee of Tokyo Medical and Dental University and the International Olympic Committee (IOC). Our study and intent to publish the data were approved by the IOC.

This study used imaging, and clinical data from the PACS and EMR collected at the Tokyo 2020 Summer Olympic Games. Medical and imaging services were open for 30 days, from the opening of the Olympic Village on 13 July 2021 until its closing on 11 August 2021. We used athlete accreditation numbers to assure athlete identity and acquire information in PACS and EMR. We treated all information with strict confidence and de-identified our medical database after the Games. Informed consent was waived because all data in our epidemiological study were anonymised and unidentifiable. We obtained approval from the IOC to use anonymised imaging and demographic data for publication. Data were collected, stored, and analysed with strict attention to data protection and athletes’ confidentiality.

### Imaging data acquisition

Diagnostic imaging was performed using the Discovery XR656HD digital X-ray system (GE Healthcare, Brazil) and two MRIs: the 1.5 T Signa Explorer and the 1.5 T Signa Voyager (GE Healthcare, Brazil) installed at the Olympic Village polyclinic. MRI images were obtained using short tau inversion recovery (STIR) or fluid-sensitive fast spin-echo sequences such as T2-weighted and proton density-weighted with fat suppression in at least two planes and T1-weighted in one or two planes, as appropriate for each anatomical location. No intravenous gadolinium was used.

We reviewed all X-rays, MRIs, and EMR databases to identify imaging-detected bone stress injuries. We excluded non-athlete patients such as team staff and cases of direct trauma as determined by clinical history in the EMR.

### Imaging interpretation

A board-certified musculoskeletal radiologist (TA, with seven years of experience in musculoskeletal imaging) and a board-certified orthopaedic surgeon (JA, with nine years of experience in musculoskeletal imaging) independently reviewed MRI and radiographic images of athletes diagnosed with bone stress injuries. The two readers were blinded to clinical history other than that a stress injury was suspected. Stress fracture was diagnosed by the presence of sclerosis, periosteal reaction, cortical thickening, and/or a fracture line at the site of pain on X-ray or MRI. Bone stress reaction was defined as an ill-defined hyperintensity area on a fluid-sensitive sequence of MRI without an apparent fracture on any imaging modalities at the symptomatic site. Two physicians recorded the location of the lesion and its Fredericson classification [15] in all cases (grade 0 = normal; grade 1 = periosteal oedema; grade 2 = marrow oedema visible on T2-weighted images only; grade 3 = marrow oedema visible on both T1-weighted and T2-weighted images; grade 4a = intracortical signal changes in multiple focal areas; grade 4b = linear region of intracortical signal changes). To evaluate the distribution of MRI grading, grade 1 and grade 2 were defined as low-grade injuries, and grades 3 and 4 were determined as high-grade injuries. If there was disagreement between the two physicians’ readings, the consensus final result was described.

### Data collection of clinical information and competition results

We recorded the following information by EMR: sex/gender, age, nationality, sport, date of injury onset, and past medical history. We obtained the competition results by EMR and from the official Olympic website. We correlated these clinical data and competition results with the imaging findings.

### Statistical analysis

EZR software version 1.55 (Saitama Medical Center, Jichi Medical University, Saitama, Japan), a graphical user interface for the R software package, was used for all statistical analyses in this study [16]. Fisher’s exact test was used to assess the incidence proportions of gender and continent of the athlete with bone stress injuries. Analysis of incidence by continent excludes the Refugee Olympic Team case. *p* < 0.05 was defined as indicating a statistically significant difference.

## Results

### Epidemiology

**Fig. 1:**
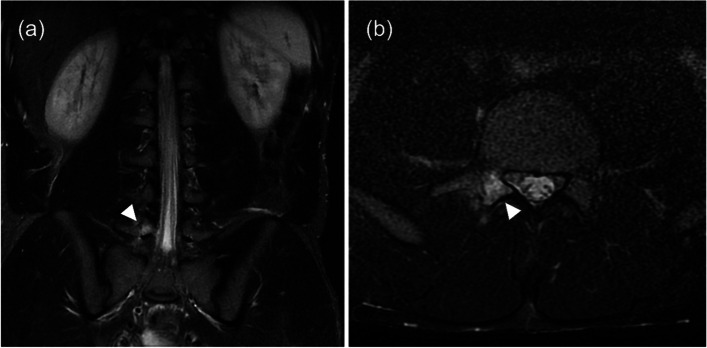
A football player diagnosed with a stress reaction of the right L5 pars interarticularis. The increased T2 signal intensity was detected on (**a**) coronal and (**b**) axial STIR images of MRI (arrows)

**Fig. 2:**
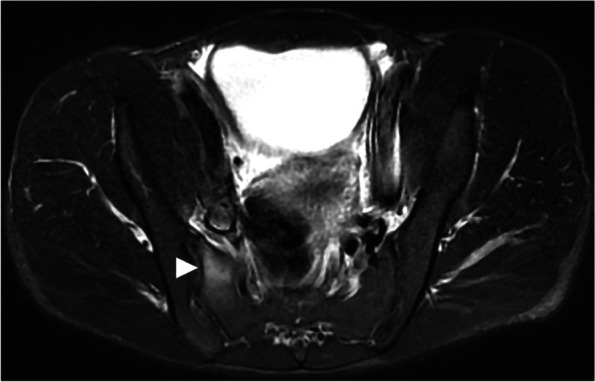
A track and field athlete with a sacral stress reaction. The increased intensity was detected at the right lateral mass of the sacrum on the axial STIR image (arrow)

There were 11,315 individual athletes (5892 males, 5423 females) representing 206 nations, territories, and principalities in competition at the Tokyo 2020 Olympic Games, during which the Olympic Village polyclinic performed 567 MRI scans and 352 X-ray scans between 13 July and 11 August 2021. The number of athletes diagnosed with bone stress injuries in the polyclinic was 29 (16 female and 13 male athletes), and nine of those had more than one bone stress injury (Figs. 1 and 2). All the athletes diagnosed with BSI underwent MRIs and seven of them underwent X-rays. There was no significant difference in the incidence of imaging-detected bone stress injury between male (0.2% of all male athletes) and female (0.3% of all female athletes) athletes (*p* = 0.55). The total number of bone stress injuries was 42 lesions (four stress fractures and 38 stress reactions), counted on a lesion-by-lesion basis (Table 1). Stress injuries were most commonly seen in track and field athletes (*n* = 13), followed by boxing, handball, rhythmic gymnastics, and triathlon (*n* = 2). There was one case each in artistic gymnastics, judo, modern pentathlon, volleyball, water polo, weightlifting, wrestling, and football. Table 2 reveals the number and location of lesions by sport. We defined marathon and race walking as road events in track and field and the remainder of track and field events as either track or field as appropriate.

**Table 1:** Location of imaging-detected bone stress injuries that counted on a lesion-by-lesion basis

Location (percentage)	Stress fracture	Stress reaction	Total
**Lower extremity total (69.0%)**	**3**	**26**	**29**
Tibial diaphysis	1	6	7
Medial malleolus	0	2	2
Medial tibial plateau	0	1	1
Femoral head	0	1	1
Femoral neck	0	1	1
Intertrochanter of the femur	0	1	1
Medial femoral condyle	0	1	1
Metatarsal bone	1	4	5
Tarsal Bone	0	8	8
Phalanx	1	1	2
**Spine (9.5%)**	**1**	**3**	**4**
Lumbar spine	1	3	4
**Upper extremity (11.9%)**	**0**	**5**	**5**
Phalanx	0	2	2
Metacarpal bone	0	1	1
Triquetrum	0	1	1
Clavicle	0	1	1
**Pelvis (9.5%)**	**0**	**4**	**4**
Acetabulum	0	1	1
Pubis	0	2	2
Sacrum	0	1	1
**Total**	**4**	**38**	**42**

**Table 2:** Sports for imaging-detected bone stress injuries in athletes at the Tokyo 2020 Olympic Games

	**Stress fracture**					**Stress reaction**				
	**Lower extremities**	Upper extremities^*^	**Spine**	**Pelvis**	**Total**	**Lower extremities**	Upper extremities^*^	**Spine**	**Pelvis**	**Total**
**Track and field**	2	0	1	0	**3**	11	0	1	1	**13**
Track	1	0	0	0	**1**	6	0	1	0	**7**
Road	0	0	0	0	**0**	2	0	0	1	**3**
Field	1	0	1	0	**2**	3	0	0	0	**3**
**Artistic gymnastics**	0	0	0	0	**0**	1	0	0	0	**1**
**Handball**	0	0	0	0	**0**	0	0	1	1	**2**
**Judo**	0	0	0	0	**0**	2	0	0	0	**2**
**Modern pentathlon**	0	0	0	0	**0**	1	0	0	0	**1**
**Rhythmic gymnastics**	0	0	0	0	**0**	6	0	0	0	**6**
**Triathlon**	0	0	0	0	**0**	2	0	0	0	**2**
**Volleyball**	0	0	0	0	**0**	2	0	0	0	**2**
**Water polo**	0	0	0	0	**0**	2	0	0	1	**3**
**Weightlifting**	0	0	0	0	**0**	0	1	0	0	**1**
**Wrestling**	0	0	0	0	**0**	0	1	0	0	**1**
**Football**	0	0	0	0	**0**	0	0	1	0	**1**
**Boxing**	1	0	0	0	**1**	0	3	0	0	**3**

^*^Lesion of the clavicle was classified as upper extremities

The median age of injured athletes was 24 years (range, 22–26) for stress fracture and 24 years (range, 18–35) for stress reaction. The continents to which the athletes’ nations belong were as follows: Africa (*n* = 13, 1.3% of all African athletes), Europe (*n* = 8, 0.2% of all European athletes), North and South America (*n* = 5, 0.2% of all American athletes), Oceania (*n* = 2, 0.3% of all Oceanian athletes), and one other (one stress reaction from the Refugee Olympic Team). There were no bone stress injuries in Asian athletes, as was also the case in London 2012 and Rio de Janeiro 2016 [13]. African nations showed a significantly higher incidence of bone stress injuries than the others (*p* < 0.001).

**Fig. 3:**
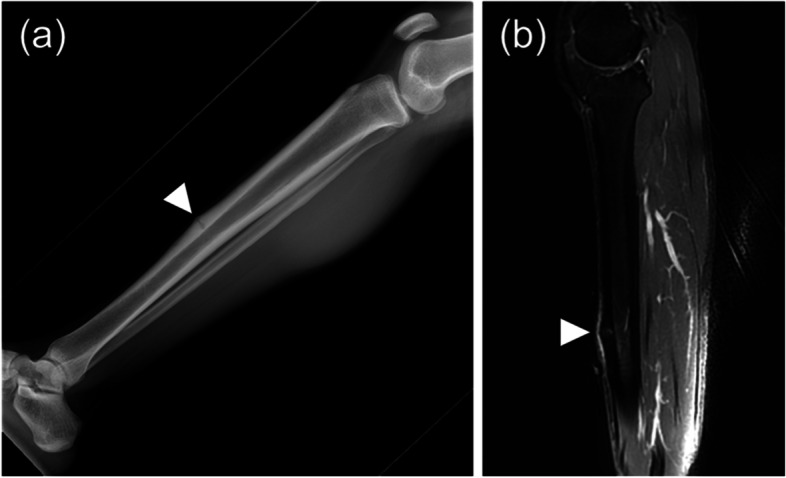
Medial tibial stress syndrome evaluated as Grade 4b in the Fredericson classification. **a** The lateral view of the lower leg X-ray demonstrated cortical thickening and fracture line of the tibial diaphysis (arrow). **b** MRI showed abnormal signal intensity in the tibial cortex and bone marrow oedema on STIR (arrow)

Medial tibial stress syndrome was present in five athletes, two of whom had bilateral lesions (Fig. 3). In the Fredericson classification, three athletes were classified as grade 3, one as grade 4a, and one as grade 4b. MRI-based grading of all athletes with bone stress injuries showed 16 with high-grade injuries and 13 with low-grade injuries.

### Medical history

Data collection from EMR revealed the chronology of bone stress injuries. Twenty-one athletes (72%) had symptoms before entering the Olympic Village. Only one of all athletes with bone stress injuries had a history of a stress fracture according to the EMR.

Table 3 lists athletes with imaging-detected bone stress injuries who did not start (DNS) or did not finish (DNF) the competitions. One athlete with a stress fracture did not finish the competition, and five athletes with stress reactions resulted in four DNF and one DNS.

**Table 3:** Detail of patients who did not start or finish the competitions

Location	Continent	Sport	Sex	Onset	Result
**Stress fracture**					
1^st^ proximal phalanx of the foot	Europe	Track and Field (Field)	F	After entering the Olympic village (During the preliminary round)	DNF
**Stress reaction**					
Talus	Africa	Triathlon	M	Before entering the Olympic village (Four weeks before MRI)	DNF
Tibia	Africa	Track and Field (Track)	M	Before entering the Olympic village (Ten days before MRI)	DNS
Calcaneus	Africa	Track and Field (Track)	F	Before entering the Olympic village (Two months before MRI)	DNF
Sacrum	Africa	Track and Field (Road)	F	Before entering the Olympic village (Four days before MRI)	DNF
Femoral neck	Europe	Track and Field (Road)	F	Before entering the Olympic village (One month before MRI)	DNF

Abbreviations: DNF, did not finish; DNS, did not start

## Discussion

### Clinical implications

During the Tokyo 2020 Olympic Games, 29 athletes and MRI scans (0.3% of all participants, 5.1% of all MRIs) at the polyclinic were diagnosed with bone stress injuries. Although there was no significant difference between males and females, bone stress injuries were most common in females (55%), in the lower extremities (66%), and among track and field athletes (45%). These results were very similar to those from Rio de Janeiro 2016, in which there were a total of 25 athletes (approximately 2% of all injuries, 0.2% of all participants) with bone stress injuries (9 stress fractures, 16 stress reactions), 72% of which were in women, 84% of which were in the lower extremities, and 44% of which were in track and field athletes [13]. Bennell et al. summarize the epidemiological characteristics of stress fractures in different sporting populations [17], indicating that bone stress injuries comprised between 0.7 to 15.6% of all athletic injuries and were most common in women and track and field athletes. These results suggest that Olympic athletes could show similar epidemiologic trends in bone stress injury as non-Olympic athletes.

This study describes the incidence of bone stress injuries by continent. It showed that athletes from the African continent were diagnosed with bone stress injuries at a significantly higher frequency. The result was insufficient to conclude a continent-specific incidence trend, as we did not consider the proportion of sports events, gender, and age. At the Rio Olympics, bone stress injuries in African athletes were second only to those in Europe, but the incidence was not evaluated [13]. Multifactorial evaluations of the incidence and risks of bone stress injuries among continents might be worth considering.

The current study provided additional details within the track and field category, separately providing data on road events (marathon and race walking). Although we did not calculate the total number of athletes in each category, about half of the track and field athletes with bone stress injuries (*n* = 6, 46%) competed in track events. Since the anatomical sites exposed to physical loading and the content and intensity of training differ among the events in track and field, it may be helpful to accumulate more detailed epidemiological information on bone stress injuries in each event of track and field.

High-intensity training is unavoidable for Olympic athletes, meaning that delayed diagnosis can result in the progression of the MRI grade of bone stress injury. According to the literature, the longer the time between symptom onset and diagnosis of the stress fracture, the longer the recovery time [18]. Hoenig et al., in their systematic review and meta-analysis [3], reported that higher MRI-based grading of bone stress injury was associated with an increased time to return to competition. Therefore, the finding that more than half of the cohort in our study had high-grade injuries emphasises the importance of prevention and early diagnosis in Olympic athletes.

We also described the details of injury onset and DNS/DNF athletes with imaging-detected bone stress injuries. Most athletes with bone stress injuries (72%) had symptoms before their arrival at the Olympic Village. Furthermore, six of 29 athletes with bone stress injuries (21%) did not start or did not finish their competitions, and four of the six athletes who withdrew had symptoms before entering the Olympic Village. In Olympic athletes, early MRI examination of symptomatic athletes, even before their arrival at the Games, could reduce the risk of withdrawal and stress injury progression. This emphasis on early diagnosis clearly extends to athletes of all levels. Although preparticipation evaluation has been performed at the athlete level regarding sports injury prevention and screening, no effective means of providing risk factors and early diagnosis for bone stress injury has been established [19]. Further investigation of early diagnostic strategies, including the validity of early MRI examination, especially at the level of elite athletes, is required.

### Limitations

Our study had several limitations. First, many of the athletes with bone stress injuries in this study did not undergo radiography; therefore we could not assess the utility of MRI compared with that of radiographs. Second, we could not obtain information on risk factors for bone stress injury such as eating disorders, menstrual dysfunction, medication use, or underlying medical conditions [20–23]. IOC policy states that no research can disrupt athlete training and competition, and therefore questionnaires regarding risk factors including eating disorders and menstrual function were not possible. Another limitation is that only athletes who visited the polyclinic and underwent radiology examinations were included in this study; potential patients with more subtle symptoms that did not warrant imaging examinations or did not visit the polyclinic were not evaluated. Finally, we relied on imaging reports in the EMR and PACS of the polyclinic to identify athletes with bone stress injuries; it is possible that some athletes with more subtle symptoms and imaging findings could have been missed.

## Conclusions

The epidemiological characteristics of bone stress injuries in the Tokyo 2020 Olympics showed a trend similar to that reported for the Rio de Janeiro 2016 Olympics.

Three-quarters of the athletes with bone stress injuries had symptoms before entering the village, and over 20% of athletes did not start or finish their competitions. Early MRI examination of symptomatic athletes, even before their arrival at the Games, may reduce the risk of stress injury progression for Olympic athletes.

## Data Availability

Unpublished data are not available for sharing. Inquiries about data availability are made by contacting the corresponding author.

## Abbreviations

DNF: Did not finish
DNS: Did not start
EMR: Electronic Medical Records
IOC: International Olympic Committee
MRI: Magnetic Resonance Imaging
PACS: Picture Archiving and Communication System
STIR: Short tau inversion recovery
